# The perceptions of players and their parents/guardians regarding the effectiveness of Cricket South Africa's Hub programme in the Western Province region of South Africa

**DOI:** 10.17159/2078-516X/2025/v37i1a18562

**Published:** 2025-06-15

**Authors:** L Sibeko, J Gray, MS Taliep

**Affiliations:** 1 Department of Sport Management, Cape Peninsula University of Technology, Cape Town, South Africa; 2 Department of Human Biology, Division of Physiological Sciences, University of Cape Town, Cape Town, South Africa

**Keywords:** sport, youth, talent, skill, training, cricket

## Abstract

**Background:**

In South Africa, the Cricket Hub development programme seeks to address the lack of access to cricketing excellence in disadvantaged communities by offering coaching, facilities, and support to the players.

**Objectives:**

To assess the effectiveness of the Hub programme through evaluating the perceptions of the players and their parents/guardians.

**Methods:**

This study used Likert-scale questionnaires to systematically capture the perspectives of the players and their parents/guardians. A total of 230 questionnaires were issued to players and their parents/guardians. There was a 46% response rate for the players and a 44% response rate for the parents/guardians.

**Results:**

The perception of the players and their parents/guardians was overwhelmingly positive across all key themes. These themes included player development, training environment and resources, match exposure, holistic impact, safety, and communication.

**Conclusion:**

The Hub programme advances the agenda of Cricket South Africa by fostering cricketing excellence in disadvantaged communities.

Most South Africans have limited access to quality sporting opportunities, which is directly linked to the country's historical socioeconomic disparities.^[1]^ Typically, communities with limited access to quality resources have fewer children participating in sports, negatively affecting the production of skilled athletes.^[2]^ Furthermore, sports development is hindered in these disadvantaged communities through crime, vandalism, and misuse of sporting facilities.^[3]^

These inequalities existing in disadvantaged communities also extend to cricket.^[4]^ Post-Apartheid Cricket South Africa (CSA) and the South African government aimed to transform cricket by promoting cricketing excellence in underserved communities.^[5]^ A significant investment was made in improving these communities’ facilities, coaching and sports administration.^[5]^ One of the programmes promoting this transformation agenda was the CSA Hub programme.^[6]^ CSA launched the Hub programme in 2013 as part of a talent development process. The Hub programme identifies and develops cricketing talent in disadvantaged communities by providing access to coaching, facilities, matches, mentorship, and life-skills programmes.^[6–7]^ The players selected for the Hub programme are generally drawn from schools where cricket is not played or prioritised.^[7]^

Millions of rands have been expended into the Hub programme, yet no formal evaluation has occurred. This research study aimed to evaluate the Hub programme by investigating the perceptions of players and their parents/guardians at the Hub.

## Methods

### Study design

This study used a quantitative design, using a 3-point Likert scale. To assess perceptions of the Hubs' effectiveness, questionnaires were distributed to all players and their parents or guardians. The questionnaires gathered feedback on various aspects, including player development, the training environment and resources, exposure to matches, holistic impact, safety, and communication.

### Questionnaire development

Questions were designed based on CSA Hub programme criteria and input from a CSA manager. The questions were then evaluated by two critical friends in the field of sport development. The questionnaire was then piloted on a parent in the Hub for further validation. Final adjustments to the questionnaire were made based on the parent's input.

### Demarcation and sample

The Western Province hosts seven CSA Hubs. One of these Hubs was not operational at the time of data collection. The questionnaires were distributed to all players and their parents/guardians at the six operational Hubs over three weeks in November 2018. A total of 230 questionnaires were distributed among players and 230 questionnaires to the parents/guardians.

### Ethics approval

Ethics approval was secured from the university's research ethics committee (CPUT Ethical Clearance Certificate number: 2018FBREC588). Players signed the assent form, and their parents/guardians signed the consent form to participate in the study.

### Statistical analysis

The description of the perceptions was reported as percentages for each question. Responses to Likert scale items were dichotomised into categories: the response "Disagree" was scored as 1, "Neutral" as 2, and "Agree" as 3. Chi-squared tests compared players' and their parents/guardians' responses. Modes related to the categories (1–3), were presented, as they provide a clearer representation of the most frequently occurring response within the dataset, making them particularly suited for interpreting Likert scale data.^[10]^ Significant difference was set at p≤0.05.

## Results

### Player demographic information

The mean age of the players was 13.0±5.4 years, with a mean time spent at the Hub of 2.8±0.5 years and a mean time playing cricket of 2.8±0.5 years (Table 1). Of the 101 parents/guardians that responded, 26 were from Hub A, 13 from Hub B, 9 from Hub C, 15 from Hub D, 24 from Hub E and 18 from Hub F.

### Players and their parents/guardians’ perception of the Hub programme

The players returned 105 (46%) of the questionnaires while the parents/guardians returned 101 (44%). However, some responders omitted some questions. Therefore, the grand total might at times, be less than 105 for the players and 101 for their parents/guardians. Tables 2–4 represent the players’ and their parents/guardians’ perceptions of the Hub programme relative to the different themes.

The modes for the players and their parents/guardians were identical across all questions (Tables 2–4), indicating that the most frequent response for all the questions was the same for both groups. Significant differences between group responses only indicate a significantly different number of agreements in the same category.

### Player development

Questions relating to player development are presented in Table 2. Players and their parents/guardians agreed that the Hub programme positively impacted the players’ skills and knowledge development. Both group of respondents also viewed that coaching and mentoring played an important role in this development. Although most players and their parents/guardians agreed that there were sufficient coaches at the Hub, there was a significant difference in the extent of their responses. Significantly more players compared to parents/guardians (32% vs 14%, p=0.006) indicated that there were not sufficient coaches at the Hub. Most players and their parents/guardians disagreed that the coaches do not interact well with the players and made the players enjoy being part of the Hub. Significantly more parent/guardians compared to the players (93% vs 73%, p=0.001) reported that the coaches contributed to the knowledge development of the player. Both groups agreed that the mentorship at the Hub was beneficial and that it further positively developed the player.

### Training environment and resources

The results of the training environment and resources at the Hub are presented in Table 3. Both players and their parents/guardians agreed that there were sufficient practice sessions, that these sessions were run effectively and were enjoyable. Although most of the players and their parents/guardians agreed that there was adequate equipment at the Hub, significantly more players compared to parents/guardians disagreed with this statement (27% vs 12%, p=0.018). Most of the players and their parents/guardians agreed that the facilities at the Hub were sufficient and were well maintained.

### Exposure to matches

Most of the players and their parents/guardians reported that there were sufficient matches at the Hub and that the matches were of a good quality (Table 3).

### Holistic impact

The holistic impact of the Hub on the player is presented in Table 4. Most of the players and their parents/guardians agreed that Hub positively affected their academic performance and contributed to social skill development and confidence. However, there were significant differences in the extent of their responses, with significantly more parents/guardians agreeing to the statements (Table 4). Although most of the players and their parents/guardians agreed that the Hub assisted the player in reaching a sporting school, the players reported significantly lower responses than the parents/guardians (42% vs 60%, p=0.011).

### Safety and communication

Although most of the players and their parents/guardians agreed that the Hub is safe and not dangerous when travelling home, significantly more players compared parents/guardians agreed with these statements (Table 4). Most players and their parents/guardians agreed that there is clear and constant communication between the players and the Hub management, but players indicated significantly lower responses than parents/guardians (79% vs 91%, p<0.001).

### Overall evaluation

Although most of the players and their parents/guardians agreed that the Hub was well organised, significantly less players compared to parents/guardians agreed to this statement (76% vs 80%, p=0.033, Table 4). Both players and parents/guardians agreed that the Hub programme is not a bad initiative nor a waste of time.

## Discussion

The aim of this study was to understand the perceptions of players and their parents or guardians regarding the Hub system implemented by Cricket South Africa. These perceptions were categorised according to the players' development, their training environment and resources, holistic impact, exposure to matches, safety, and communication. Despite some notable differences in responses between the players and their parents or guardians, the modes for all responses were the same. This indicates that most participants in both groups either agreed or disagreed with the statements.

### Player development

Players and their parents/guardians agreed that the Hub programme positively impacted the players’ skills and knowledge development. Both groups of respondents also viewed coaching and mentoring as having played an important role in this development. These results indicate that the Hub programme effectively provided coaching and mentoring to the young cricketers to develop their game. The coaches at the Hubs play a multifunction role. The coach can fulfil the role of a technical assistance, motivator, leader, mentor, and a role model for the cricketers to follow both on and off the field of play.^[11]^ A positive coach-athlete relationship is an important component of success. This creates effective communication, motivation, trust, and support, which are important for both skill development and athlete well-being.^[12]^ Coaches were further perceived to make the players' experiences enjoyable, which underscores the important role coaches play in creating a fun and engaging environment for the young players. Enjoyment and positive experiences during training are associated with overall athlete retention, improved performance, and satisfaction.^[13]^ Coaches also play an important role in improving players’ understanding and tactical skills. By expanding players’ skills and knowledge, coaches contribute to their overall development.^[11]^ Coaching players at the Hub enables coaches to build trusting relationships with cricketers, taking on the role of mentor and coach. The importance of mentoring in the South African transition context cannot be overstated.^[14]^ This aligns with the broader objectives of the CSA transformation policy, which seeks to provide inclusive and equal opportunities for all individuals in cricket, particularly those from historically disadvantaged backgrounds.^[14]^ The coach is probably the most influential person influencing the development of the player at the Hub, and CSA must make ensure the best personnel hold these positions.

The success of a sports programme relies on adequate, quality coaching that supports sports development and assists athletes in progressing to an elite level. There is concern that there may be too few coaches relative to the number of players at Hub. The optimal coach-to-player ratio is suggested to be one adult coach for every 8–10 children (aged over eight years). Although we are uncertain about the coach-to-player ratio at the Hubs, CSA should ensure that all Hubs are suitably equipped for coaches to engage optimally with the players.

### Training environment and resources

Players and their parents/guardians felt that there were sufficient practice sessions, which were run effectively and enjoyable. Adequate practice sessions allow cricketers to refine their techniques, build their physical fitness, and develop a deeper understanding of the game. Enjoyment and engagement are key factors in promoting sustained participation and player development^[16]^ and key to the success of the Hub programme. Although most of the participants agreed that there were adequate equipment and facilities at the Hub, it is concerning that 27% of the players felt that the equipment was not adequate. This suggests that there may be a need for improvement in the provision of equipment to better support player development. Good facilities foster skill development, creating a conducive training environment and ultimately enhancing participation and performance.^[17]^ We recommend a review of the facilities at the Hubs in the Western Province.

### Exposure to matches

There was a perception that the Hub provided sufficient matches and that these matches were of a good standard. The Hub thus provides a viable means of providing better opportunities for cricket participation and development of the game in the local areas. These findings correlate with reports that regular participation during a season can assist in maintaining and improving players’ physical and skill performances.^[18]^ In addition, this improvement can result in some players garnering success in that specific talent pathway and attaining selection in senior or provincial teams.

### Holistic impact

There was a perception that the Hub programme had a positive effect on academic performance, social skills and self-confidence. The Hub runs 12 months of the year and provides physical activity through participation in cricket two to three days a week. This consistent exposure to physical activity aligns with previous research, suggesting that increasing physical activity levels can significantly improve grades.^[19]^ Players participating in the Hub are also aided academically through the CSA School in the Box initiative. This is an intervention offered at some Hubs where tutors are assigned in an after-school programme aimed at providing the identified cricketers in the community support with their education. Some players were able to transfer to sporting schools because they participated in the Hub programme. This is because they received a sports scholarship to attend these sporting schools. Another reason could be because these schools recognised the talent of the Hub player and made the process of acceptance into the school easier. Most South African senior international players come from these sporting schools.^[20]^ The long history of sporting culture and excellence, backed up by the state-of-the-art equipment, quality coaching and facilities at these schools, make them a breeding ground for international cricket players.^[20]^ Parents/guardians in disadvantaged communities struggle to afford the high fees associated with schools with a sport focus and access to these schools through the Hub programme is advantageous.

The results of this study indicate that the Hub programme had a significant positive impact on the cricketers' social skills and confidence development. The Hub also promoted self-confidence in the players. Enhancing confidence is a significant aspect of youth sports development, as it impacts their performance on the field and overall well-being.^[2]^

### Safety and communication

Most players and their parents/guardians agreed that participation at the Hub is safe. Player safety is a fundamental aspect of youth sports programmes, particularly in South African, where safety and security can be an obstacle to sport participation. It is crucial that participants feel protected and free from potential harm or risks, by implementing safety protocols and measures to protect the well-being of participants.^[21]^ Many of the Hubs are in areas renowned for crime and having the Hub as a safe area to participate is crucial to the long-term sustainability of the sport in these communities.

The communication channels established within the Hub programme were reported to be effective, ensuring that the players receive clear and consistent information. Effective communication between parents/guardians, and coaches can build trusting relationships supporting a strong sport culture like the Hub programme.^[22]^

### Overall evaluation

Most players and their parents/guardians widely regard the Hub programme as a valuable initiative with the potential to enhance talent development significantly. Parents/guardians have expressed a positive outlook on the programme, perceiving it as a prudent investment of their children's time. Bullock et al.,^[23]^ argued that cricket programmes of this nature are important in imparting knowledge, extending support, and providing valuable learning avenues, all of which collectively contribute to cultivating a more constructive and encouraging environment for developing cricketers. This shared sentiment among parents/guardians and players reflects a comprehensive understanding of the pivotal role played by the Hub programme. It underscores the intrinsic value it bestows upon the holistic development of cricket.

### Strengths and limitations

There are few strengths and limitations of the study that we would like to highlight. In respect to the strengths, this is the first published study to investigate the effectiveness of a CSA development initiative like the Hub programme. The study provides key stakeholders, like the players and parents/guardians, an opportunity to express their view on the programme's effectiveness. Furthermore, key findings suggest the programme is mainly effective in achieving the goals set out by CSA.

The study's findings need also to be interpreted with respect to its limitations. Firstly, only 46% of the players and 44% of their parents/guardians returned the questionnaires, which can affect the generalisation and representation of the results. Secondly, limited statistical analyses were performed because of the low response rate at certain Hubs. Therefore, a rigorous comparison between Hubs was not possible. Finally, perceptual studies of this nature are limited to the views of the participants and no objective outcomes were measured.

## Conclusion

The Hub programme is valuable in South Africa's cricket transformation agenda. It provides opportunities for young cricketers from disadvantaged communities to participate in the sport and develop their skills. The positive perceptions of players and their parents/guardians indicate the value and significance of the programme in fostering cricket development.

The Hub programme has demonstrated its potential as an effective platform for talent development in cricket, addressing the needs and aspirations of young cricketers from disadvantaged communities. By continuing to prioritise skill development, knowledge acquisition, mentorship, and holistic player development, CSA can further enhance the impact of the Hub programme and contribute to the long-term growth and success of cricket in the country. Furthermore, the goals CSA set out to accomplish through the Hub programme have been met during its implementation.

Future research can delve deeper into specific areas identified for improvement, such as equipment, adequate facilities and facility maintenance and coach-to-player ratios. Additionally, investigating the long-term outcomes of the Hub programme, such as players' cricketing careers and overall well-being, would provide valuable insights into the programme's effectiveness.

## Figures and Tables

**Table 1:** Number of players and parents/guardians (PG) per Hub, along with player demographic details, including player age, years of participation at the Hub, and prior cricket experience

	Player (n)	PG (n)	Age (years)	Years at the hub	Cricket experience (years)

Hub A	17	24	13±3.6	3±0.6	3±0.4
Hub B	11	11	13±6.8	3±0.3	3±0.4
Hub C	20	9	13±4.1	2±0.4	3±0.5
Hub D	13	15	13±8.6	3±0.2	2±0.9
Hub E	25	25	13±6.4	3±0.6	3±0.4
Hub F	19	18	15±3.4	3±0.6	3±0.4

Mean ± SD			13±5.4	2.8±0.5	2.8±0.5

**Table 2:** The perception of the players (P) and their parents/guardians (PG) on the player development at the Hubs.

Player development	Disagree (%)		Neutral (%)		Agree (%)		Mode (p)	Mode (p/g)	p-value

	P	PG	P	PG	P	PG			
				
2.1 The Hub has contributed to my/my child’s skill development	5	2	5	6	95	92	3	3	0.326
2.2 The Hub has contributed to my/my child’s knowledge of the game of cricket	5	6	3	8	92	92	3	3	0.281
2.3 There are too few coaches at your Hub	49	69	20	17	32	14	1	1	0.006*
2.4 The coaches do not interact well with the players	81	85	8	5	11	10	1	1	0.704
2.5 The coaches make you/your child enjoy being part of the Hub	1	2	2	3	97	95	3	3	0.734
2.6 The coaches contribute to my/my child’s skill development	0	1	1	6	99	93	3	3	0.084
2.7 The coaches do not contribute to my/my child’s knowledge development of the game	73	93	8	3	19	4	1	1	0.001*
2.8 The mentorship programmes at your Hub are beneficial to the cricketer	6	4	16	8	78	88	3	3	0.180
2.9 I have developed as a result of the mentorship at the Hubs	5	5	9	10	86	85	3	3	0.965

Data are presented as percentages, modes and Chi-squared values for the differences between the groups.

* p-value ≤ 0.05.

Total (%) might not equal 100 due to rounding off.

**Table 3:** The perception of the players (P) and their parents/guardians (PG) relating to the training environment, resources and exposure to matches at the Hubs.

Training environment and resources	Disagree (%)		Neutral (%)		Agree (%)		Mode (p)	Mode (p/g)	p-value

	P	PG	P	PG	P	PG			
				
3.1 There are enough practice sessions per week	13	12	10	10	77	78	3	3	0.957
3.2 The practice sessions are being run effectively	7	6	11	16	82	78	3	3	0.631
3.3 Practice sessions are not enjoyable	75	88	8	4	18	8	1	1	0.062
3.4 The Hub has adequate cricketing equipment to support my/my child’s development	27	12	20	18	52	70	3	3	0.018*
3.5 The Hub has adequate facilities to support my/my child’s development	18	15	19	23	62	62	3	3	0.693
3.6 The facilities at your Hub are not well maintained	55	53	25	34	19	14	1	1	0.326

**Exposure to matches**									

3.7 Players played a sufficient number of matches at the Hub	22	13	18	18	60	69	3	3	0.197
3.8 The quality of the Hub matches is poor	75	76	17	16	8	6	1	1	0.987

Data are presented as percentages, modes and Chi-squared values for the differences between the groups.

* p-value ≤ 0.05.

Total (%) might not equal 100 due to rounding off.

**Table 4:** The perception of the players (P) and their parents/guardians (PG) relating to the holistic impact on the player, safety, communication and overall evaluation of the Hubs.

Training environment and resources	Disagree (%)		Neutral (%)		Agree (%)		Mode (p)	Mode (p/g)	p-value

	P	PG	P	PG	P	PG			

**Holistic impact**									

4.1 Being part of the Hub has positively affected my/my child’s academic performance at school	22	9	21	14	56	77	3	3	0.007*
4.2 The Hub programme assisted me/my child to move to a sporting school	39	20	20	20	42	60	3	3	0.011*
4.3 The Hubs have contributed to the development in my/my child’s social skills	3	17	16	21	81	62	3	3	0.001*
4.4 The Hub has contributed to the development in my/my child’s confidence as a person	4	2	7	12	89	86	3	3	0.368

**Safety and communication**									

4.5 I fear for my safety at the Hubs	89	54	4	20	7	27	1	1	<0.001*
4.6 It is dangerous for me to come to the Hub	90	84	7	3	3	13	1	1	0.017*
4.7 There is clear and constant communication between the players and management of the Hub	8	2	14	8	79	91	3	3	<0.001*

**Overall evaluation**									

4.8 The Hub programme is well organised	5	8	19	11	76	80	3	3	0.033*
4.9 The Hub programme is a bad initiative	97	94	3	6	0	0	1	1	0.271
4.10 I feel that the Hubs are a waste of my time	98	92	2	6	0	2	1	1	0.188

Data are presented as percentages, modes and Chi-squared values for the difference in the two groups.

* p-value ≤ 0.05.

Total (%) might not equal 100 due to rounding off.
